# Wheat seeds exposed to heat during formation can germinate at high temperatures

**DOI:** 10.3389/fpls.2025.1539926

**Published:** 2025-03-28

**Authors:** Sachiko Toda-Matsunaga, Yusuke Toda, Ryosuke Mega, Shota Tadano, Megumi Alyza, Yuji Yamasaki, Kinya Akashi, Hisashi Tsujimoto

**Affiliations:** ^1^ United Graduate School of Agricultural Sciences, Tottori University, Tottori, Japan; ^2^ Graduate School of Agricultural and Life Sciences, The University of Tokyo, Tokyo, Japan; ^3^ Graduate School of Science & Technology for Innovation, Yamaguchi University, Yamaguchi, Japan; ^4^ Faculty of Agricultural Sciences, Tottori University, Tottori, Japan; ^5^ Arid Land Research Center, Tottori University, Tottori, Japan

**Keywords:** bread wheat, seed germination, high temperature, heat shock protein, fatty acid, metabolome

## Abstract

A capacity for reliable germination under elevated temperatures is a crucial factor in maintaining the stability of bread wheat (*Triticum aestivum*) yields in the context of climate change. Although the environment of the parent plant during growth is a known factor affecting seed germinability, the effect of this environment on the heat tolerance of wheat seeds has not been investigated in detail. To investigate the effect of exposure to high temperatures during growth, plants were exposed to 38°C at various growth stages. In germination test, seeds exposed to heat during their development had better heat germinability than the control. On the other hand, high temperatures before the seed development stage resulted in a lower temperature germinability compared to the control. To identify critical factors that altered heat germinability, we analyzed heat shock protein expression, fatty acid composition, and metabolite profiles. High-temperature treatment during seed formation increased the expression of heat shock proteins and reduced the degree of unsaturation of fatty acids in the seeds, which may enhance the ability of seeds to survive and germinate at high temperatures. There was a significant treatment effect on the overall metabolite content of the seeds. PLS regression analysis using the germination test results revealed that taurine, thymidine, beta-alanine, sinapic acid, and deoxyguanosine contributed significantly to germination rate. These findings suggest that the combined influence of these metabolites may play a role in acquiring seed germinability under high-temperature conditions during the growth period of the parent plants. These findings suggest potential components of a molecular mechanism in bread wheat that is triggered by high temperature during seed development and results in the acquisition of heat germinability.

## 1 Introduction

According to global warming scenarios, it is anticipated that global surface air temperature increases will surpass 1.5°C by 2030 in comparison to the period between 1850 and 1900 (Intergovernmental Panel on Climate Change (IPCC), 2021). The prospect of feeding the world’s future global population is a significant concern in this context of climate change. High-temperature environments can limit crop productivity; it is therefore imperative to develop new strategies to stabilize it in such environments. In particular, bread wheat (*Triticum aestivum*, 2n = 6x = AABBDD), a widely cultivated crop worldwide, is less resilient to elevated temperatures than other major cereal crops. Elevated temperatures can decrease wheat growth and yield, with heat stress during the seedling and vegetative stages being a particular concern (Matsunaga et al., 2021b; Zhao et al., 2017).

Wheat is usually cultivated by direct sowing of seeds into the field without prior manipulation to facilitate growth to the seedling stage in cell trays or by other means. Therefore, germinability directly affects the optimal seeding rate, which is in turn a crucial determinant of final yield (Hu et al., 2018). However, seed germination is suppressed by elevated temperatures (Lal et al., 2021). In some regions, such as Africa, wheat must germinate at high temperatures, and global warming may exacerbate them (Iizumi et al., 2021). A consistently high germination rate at high temperatures would be of considerable benefit. To date, the application of heat or drying treatments to seeds following harvest has been identified as a principal approach for enhancing germination rates (Paparella et al., 2015).

The impact of the plant growth environment on the degree of seed dormancy has been documented in wide range of species (Fenner, 1991), but how the growth environment affects seed germination stress tolerance remains largely unaddressed. Heat exposure (25°C) during grain filling of barley alters DNA methylation levels of phytohormone-related gene promoters, resulting in transcriptional changes and increased seed germination at 22°C (Sakai et al., 2022), but seed germinability at high temperature was not tested.

The aim of this study was to ascertain the effect of heat treatment during growth of wheat plant on germinability of their seeds at high temperature. This study was designed to address three specific objectives.

The first objective was to evaluate the impact of heat exposure during the growth period on germinability. In our previous studies, the effects of heat exposure at different growth stages from seedling to maturity, on yield-related traits and metabolite accumulation, were evaluated (Matsunaga et al., 2021a, 2021b). Heat exposure at GS2 reduced plant height and grain number, while heat at GS3 shortened the grain formation period and decreased grain weight. Consequently, plants exposed to high temperatures at GS2 and GS3 showed a decrease in yield. However, heat exposure at GS1 increased grain weight without affecting yield. The application of heat treatment at three distinct growth stages (from germination to tillering, from tillering to flowering, and from flowering to maturity) revealed that (1) seed formation is influenced by the timing and duration of senescence initiation, (2) the high temperature during growth results in a diverse range of seed morphologies, and (3) prolonged exposure to elevated temperature during the growth period impedes the progression of senescence during seed formation, thereby facilitating high-temperature acclimation (Matsunaga et al., 2021b). The present study employed the identical heat treatments to ascertain the impact of heat exposure during growth on germinability.

The second objective was to elucidate the causal changes in the internal molecular behavior of the seeds that contribute to their germinability under high temperatures. This part of the study focused on heat shock proteins (HSPs) and fatty acids. HSPs are a class of proteins that protect other proteins from thermal denaturation. Among the many HSPs, HSP70 and HSP17.6 were treated in this study. HSP70 is one of the most abundant HSPs in eukaryotic cells and serves as a chaperone involved in the maintenance of cellular protein homeostasis, as well as a regulator of the heat shock response (Hahn et al., 2011; Berka et al., 2022). A small HSP, HSP17.6, has been identified in *Arabidopsis thaliana* as a heat stress-inducible factor that enhances tolerance to subsequent heat stress (Rikhvanov et al., 2007). Some small HSPs impart tolerance to environmental stress during germination (Waters and Vierling, 2020). The contents of HSP70- and HSP17.6-like proteins in seeds increase as a consequence of heat stress during the seed formation period (Blumenthal et al., 1998). Based on these reports, an investigation into the expression of these HSPs seems crucial for understanding how germinability can be acquired in a heated environment.

Fatty acids constitute a principal component of biomembranes. To maintain biomembrane functionality, the lipid bilayer must retain a suitable fluid state, but this state is directly influenced by external temperature conditions. Elevated temperatures increase membrane fluidity, which can lead to membrane damage and disruption of biological functions (Los and Murata, 2004). The fluidity of a biomembrane is dependent upon the composition of the lipids. The ratio of unsaturation to saturation of the hydrocarbon chains is a significant factor in this regard (Niu and Xiang, 2018). The absence of double bonds in saturated fatty acids results in a straighter structure, which in turn leads to a denser arrangement and consequently lower membrane fluidity. Wheat plants growing in high-temperature environments increase their content of saturated fatty acids which are relatively stable in high-temperature environments (Narayanan et al., 2016). Therefore, we investigated the seed content of major fatty acids (palmitic and stearic, oleic, linoleic, and linolenic acid), which are the most abundant in plants (He et al., 2020), to elucidate the manner in which the molecular structure of wheat seeds undergoes alteration in response to exposure to high temperature.

The third objective of this study was to identify other metabolites that may affect germinability at high temperatures. Several plant hormones are implicated in germination (Miransari and Smith, 2014). Studies on *A. thaliana* have indicated that abscisic acid (ABA) and auxin (indole-3-acetic acid, IAA) play a role in germination at high temperatures (Tamura et al., 2006). It is also possible that other metabolites in addition to those already mentioned are associated with germinability under elevated temperature conditions. Accordingly, the metabolites associated with acquisition of germinability in elevated temperature conditions were investigated, to elucidate possible causal metabolic pathways.

## 2 Materials and methods

### 2.1 Plant material and growth conditions

**Figure 1:**
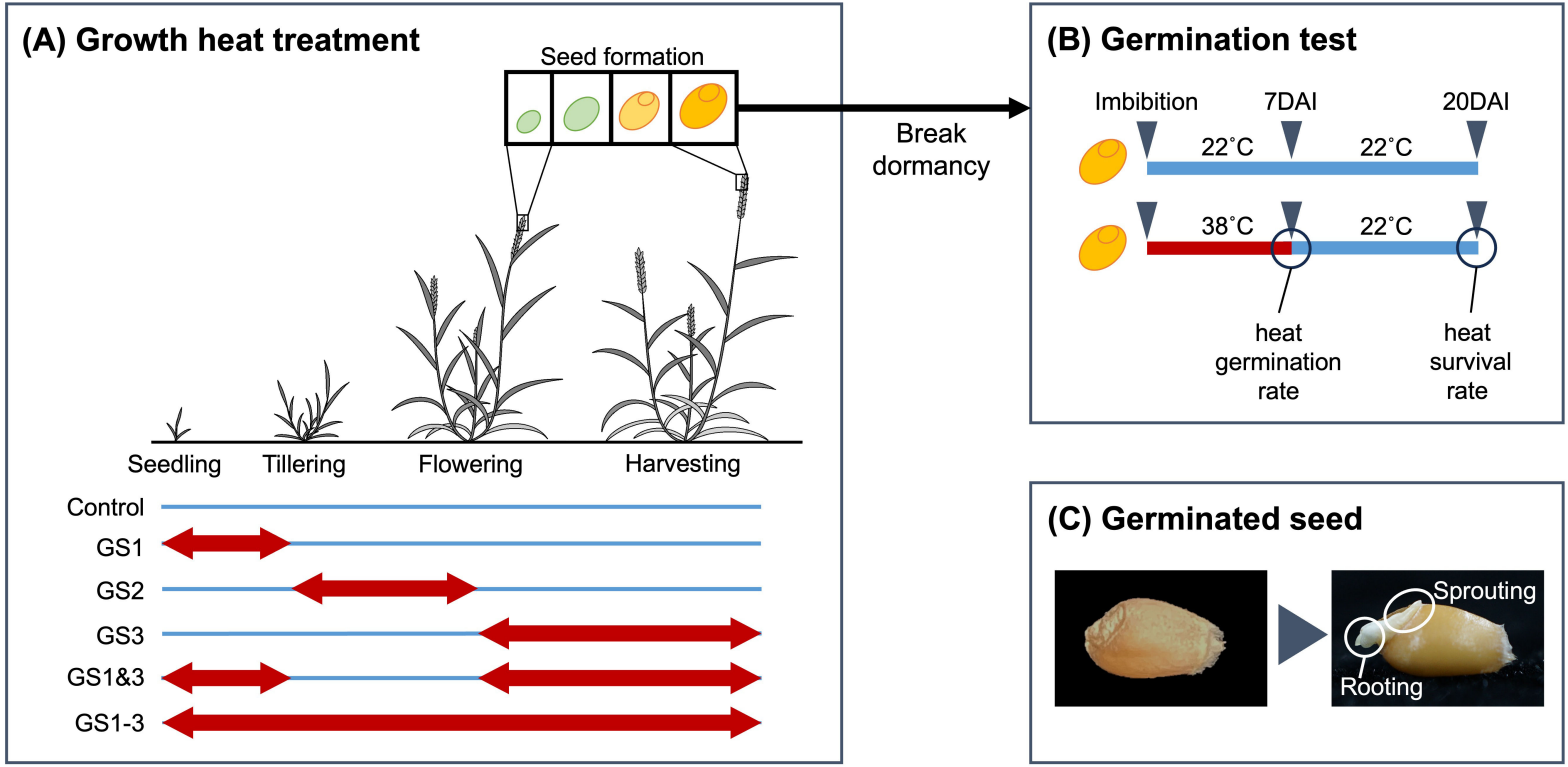
Heat exposures and germination. **(A)** Heat treatments during plant growth. Wheat plants were grown at 22°C day/18°C night (blue lines) or 38°C day/18°C night (red arrows). **(B)** Germination test. Seeds in the high-temperature treatment were moved to the control temperature at 7 d after imbibition (DAI) and held there until 20 DAI. **(C)** Germination (a control seed as an example). Note that both sprouting and rooting are observable.

Facultative spring wheat cultivar “Norin 61”, adopted as the representative Japanese bread wheat cultivar in the 10+ Genome Project (Walkowiak et al., 2020), was used for this study. Plants were cultivated under five distinct heat treatment regimes plus a control (**Figure 1A**). Four regimes plus a control were as in previous study (Matsunaga et al., 2021b), i.e., C, control; GS1, heat treatment from two-leaf unfolded to tillering (Z12–Z19 in the Zadoks decimal growth scale [Acevedo et al., 2002; Zadoks et al., 1974]); GS2, heat exposure from tillering to flowering (Z20–Z61); GS3, heat exposure from flowering to full maturity (Z62–Z95); GS1–3, heat exposure during the entire growth period. A novel treatment (GS1&3, heat exposure from two-leaf unfolded to tillering and from flowering to full maturity) was added. The purpose of this treatment was to ascertain whether the heat acclimation acquired with GS1 could mitigate the damage caused by GS3. At the end of these cultivation, seeds were harvested from six plants per treatment to assess their germination potential. Harvested seeds were stored for more than one year at room temperature (20–30°C) with a humidity level maintained below 20%.

### 2.2 Germination test

The germinability of seeds collected from plants subjected to different heat treatments was evaluated. The seeds were sterilized by immersion in 70% ethanol for one minute, followed by 10% hypochlorous acid for one minute, and then rinsed three times with distilled water. A filter paper was placed on a 90 mm plastic Petri dish, distilled water was added, and 10 sterilized seeds were arranged on top of the wetted filter paper. Although the number of seeds was relatively few, a simulation study revealed that the result of this experimental design was reproducible (**Appendix**). To prevent desiccation during water absorption (imbibition) and subsequent germination, the Petri dishes were covered with lids and sealed with plastic tape. Twenty seeds were tested for each of 6 parent plants, thus a total of 12 Petri dishes were prepared for each treatment.

Ten seeds per parent plant (six Petri dishes per treatment) were maintained in the dark either at 22°C for 20 d (control) or at 38°C for 7 d and then at 22°C for 13 d (heat treatment) and were observed daily (**Figure 1B**). The filter paper was kept moist by adding water as required. These conditions were established using an incubator (TG-180WLED-5LE, Nippon Medical & Chemical Instruments, Osaka, Japan). The purpose of transferring the seeds to 22°C for 13 d was to investigate whether seeds that did not germinate after exposure to high temperature were in a state of secondary dormancy or had lost their germinability. Germination date was defined as the time at which the seeds began to sprout and develop roots (**Figure 1C**). The number of germinated seeds was recorded at 24-hour intervals, and percentages were calculated as germination rate (germinated seeds/total seeds). For seeds exposed to the high-temperature treatment, the germination rate on day 7 was designated the heat germination rate, and the germination rate on day 20 after high-temperature imbibition for 7 d followed by normal-temperature imbibition for 13 d, was designated the heat survival rate. Significances of differences of germination rate among treatments were tested using analysis of variance and Tukey’s range test. We did not conduct any chemical assays to assess seed viability, such as the tetrazolium test, after our germination test because it was evident that the seeds had lost their germinability, as indicated by the leakage of internal tissues from the seeds.

### 2.3 Immunoblot analysis

Dried seed was separated into embryo and endosperm using a razor blade. Each portion was ground and mixed in 600 μL of extraction solution [125 mM Tris-HCl (pH 8.8), 1% (w/v) sodium dodecyl sulphate (SDS), 10% (v/v) glycerol, and 20 mM DL-dithiothreitol, 1x concentration of cOmplete™ Protease Inhibitor Cocktail (Roche)] on ice. The total protein content of the supernatant was determined by Bradford protein assay. Proteins (2.5 µg per lane) were separated by SDS-polyacrylamide gel electrophoresis (SDS-PAGE) using 12% (m/v) gels (Bio-Rad, California, USA, cat#1610175) followed by colloidal Coomassie blue staining (Dyballa and Metzger, 2009) to observe expression pattern of total proteins in tissues and western blot analysis for expression of heat shock proteins. The membrane was blocked with EveryBlot Blocking Buffer (Bio-Rad, cat# 12010020) for one hour at room temperature, and then incubated with anti-HSP17.6 (Rabbit-Poly, Agrisera, Vännäs, Sweden, cat# AS07 254) or anti-HSP70 (Rabbit-Poly, Funakoshi, Tokyo, Japan) for one hour at room temperature. Total proteins in SDS-PAGE gels for western blot analysis were electrophoretically transferred to PVDF membrane (Pall BioTrace, 0.45 µm pore size) 2 hours at 400 mA at room temperature, using the following electrophoresis buffer (25 mM Tris, 192 mM glycine, 20% methanol (vol/vol)). The membrane was then incubated in PBST mixed with a secondary antibody, goat anti-rabbit IgG (GAR) conjugated peroxidase (KPL Peroxidase-Labeled Antibody To Rabbit IgG (H+L), Sera Care), for 45 minutes at room temperature. Peroxidase activity of secondary antibody was chemiluminescent using Chemi-Lumi One Super (Nacalai Tesque, Kyoto, Japan, cat# 02230) and was photoimaged using LAS4000EPUVmini-TU (FUJI FILM, Tokyo, Japan).

### 2.4 Analysis of fatty acid composition

The fatty acid composition of seeds, which reflects differences in the internal molecular structure of the seeds that have acquired heat germinability, was analyzed (Larkindale and Huang, 2004). For each growth treatment, embryos and endosperms were separated from five seeds using a razor blade and then ground separately. The samples were mixed in 1.5 mL of a solution of t-butyl methyl ether: methanol (2:1 (v/v)) plus 0.01% (v/v) butylated hydroxyl toluene (BHT). The supernatant was collected and dried in a vacuum dryer. The fatty acids in the dried material were methylated using a fatty acid methylation kit (Nacalai Tesque, cat# 06482-04) and then purified using a methylated fatty acid purification kit (Nacalai Tesque, cat# 06483-94).

The purified fatty acids methyl esters were quantified using gas chromatography–flame ionization detector system (GC-FID; GC-4000, GL Sciences, Tokyo, Japan) and a DB-WAXetr column (0.25 mm × 30 m, 0.25 μm, Agilent Technologies, CA, USA). The temperature program was: 60°C for 3 minutes, increase at 25°C minute^−1^ for 7.6 minutes, 250°C for 15 minutes, decrease at 40°C minute^−1^ for 4.75 minutes, 60°C for 5 minutes. Methyl esters of five typical fatty acids (palmitic acid, C16:0; stearic acid, C18:0; oleic acid, C18:1; linoleic acid, C18:2; linolenic acid, C18:3) were detected using a flame ionization detector. The contents of the five fatty acids were calculated by comparing the retention time and peak area of samples and the standard compounds (Fujifilm Wako Pure Chemical, Osaka, Japan). The fatty acid ratios were determined as the ratio of each fatty acid to the total amount of the five fatty acids and expressed as a percentage.

### 2.5 Metabolite analysis

To evaluate the metabolic differences between the samples, we investigated seed metabolite accumulation and the effect of the parental growth environment on it. A total of five seeds from each of six plants from each treatment were harvested and subsequently collected for later analysis. Five seeds in a sample collection tube represented one replication; six replicates were evaluated for each treatment. The samples were pulverized using a Multi-beads Shocker ^®^ (Yasui Kikai, Osaka, Japan), and then stored in a desiccator at room temperature. Extraction of metabolites from the samples was previously described (Matsunaga et al., 2021a). Specifically, dry powdered sample (20 mg) was extracted with 200 μL (10-fold volume) of 80% methanol (LC/MS grade: Fujifilm Wako, cat# 138-14521) for 24 hours at room temperature in the dark. The extract was centrifuged at 15,000 rpm for 5 minutes at 4°C. This supernatant was centrifuged a second time under the same conditions to remove any residual insoluble material.

A total of 76 metabolites were quantified from the final supernatant using a triple quadrupole liquid chromatography–tandem mass spectrometry (LC-MS/MS) system (Agilent 6420, Agilent Technologies) and a Discovery HS-F5 column (2.1 × 250 mm, 5 μm, Sigma-Aldrich, MO, USA). The metabolites were identified by multiple reaction monitoring (MRM) analysis. The product ions used for the characterization of each metabolite are presented in **Supplementary Table S1**. Standard curves for the metabolites were constructed using mixtures of metabolite standards at a range of concentrations (0, 0.08, 0.4, 2, and 10 ppm). Compounds that exhibited similar molecular weights or retention times were not included in the same mixture. The mobile phase consisted of 0.1% formic acid (LC/MS grade: Fujifilm Wako, cat# 067-04531) and acetonitrile (LC/MS grade: Fujifilm Wako, cat# 018-19853) as A and B solutions, respectively. A gradient flow consisting of the following four A:B ratios was employed: (1) 100:0 for a duration of 2 minutes, (2) 75:25 for 8 minutes, (3) 65:35 for 4 minutes, and (4) 5:95 for 3 minutes. Only the metabolite peaks with a high signal-to-noise ratio were evaluated. The accuracy of the metabolite quantification was validated by checking whether the relative standard deviations (RSD) within the replications were less than 30%.

### 2.6 Statistical analysis of metabolite content

The metabolite contents per seed were subjected to statistical analysis to identify and illustrate the principal factors influencing the effects of the heat treatments during growth. Initially, outliers were removed using the following steps: (1) calculation of Z-scores for seed contents across all treatments, (2) identification of tentative outliers, defined as values with the greatest absolute value, (3) re-calculation of Z-scores with the tentative outliers excluded, (4) designation of values exceeding 4 as outliers, and (5) repeated application of steps 2–4 until no new outliers were identified. As only zero or one outlier was identified for each metabolite, these steps were repeated a maximum of twice.

Subsequently, an adjustment method was sought to remove the effect of embryo size from the metabolite content data, given that the majority of metabolites were present in embryos (Han et al., 2017). Since embryo weight data could not be obtained, the metabolite contents were adjusted to counteract the overall trend resulting from the heat treatments during plant growth. Fold changes (FCs) were calculated for each metabolite in each treatment, using the metabolite contents per seed divided by the corresponding content in the control condition. Subsequently, the FC of each metabolite was divided by the mean FC for the treatment. This adjustment was predicated on the assumption that the expression of the majority of metabolites was unaltered by the treatments. The FC or log_2_(FC) values were compared as metrics of metabolite content in subsequent analyses, based on their distributions and variances. On the basis of the data presented in the Results section, the log_2_(FC) of the amount adjusted by the third method was used in the subsequent analyses.

To visualize the treatment effects on seed content of all metabolites, principal component analysis (PCA) was applied. Prior to analysis, the log_2_(FC) of the seed contents were scaled with standard deviations of differences from the within-treatment mean. Subsequently, probabilistic PCA, implemented as the “pca” function in the R package “pcaMethods” (version 1.82.0), was used to address the issue of missing values. The options were set as “method = ‘ppca’” and “nPcs = 10.” The metabolites with absolute loading scores exceeding their standard deviation were identified as those significantly affecting each principal component.

To assess the impact of heat treatments during growth on seed metabolite contents, the log_2_(FC) values of each treatment were statistically compared to those of the control using Dunnett’s method, a many-to-one comparison method implemented in the R package “multcomp” (ver. 1.4-17).

Partial least squares (PLS) regression was used to quantify the contributions of the metabolites to the germination rate. PLS regression, a variant of multiple regression, calculates latent variables with the greatest covariance from each of the explanatory and target variables, thereby rendering it robust to multicollinearity. Given the prevalence of high correlations in metabolite datasets, PLS regression is deemed an appropriate method when using metabolite data as explanatory variables (Gromski et al., 2015). In this study, the log_2_(FC) of the metabolite data and the germination rates were used as the explanatory and target variables, respectively. The regression models were estimated independently for the heat germination rate and the heat survival rate. The numbers of latent variables in the PLS regression models was 1 for the heat germination rate and 9 for the heat survival rate. These values were selected from the range of 1–10 using a 10-fold cross-validation method, which was repeated 10 times. In the cross-validation, the prediction errors were evaluated using a misclassification rate. The cross-validation was conducted using the “cv.plsRglm” function in the ‘plsRglm’ R package (version 1.3.0), with the following parameters: nt = 10, modele = “pls-glm-logistic”, K = 10, and NK = 10. Once the number of latent variables (nt) had been determined, the regression coefficients were estimated using the plsRglm function with the following parameters: nt = nt, modele = “pls-glm-logistic”, scaleX = TRUE.

## 3 Results

### 3.1 Germination rate

**Figure 2:**
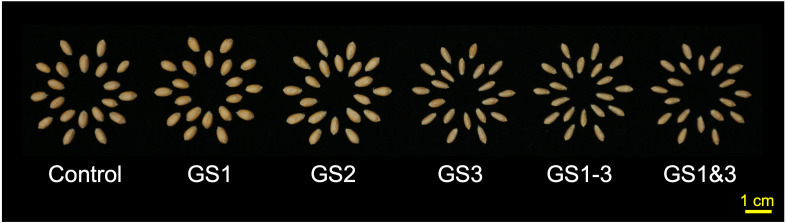
Morphology of mature seeds from plants exposed to high temperature during various growth stages. Treatment identifiers are as shown in **Figure 1**.

**Figure 3:**
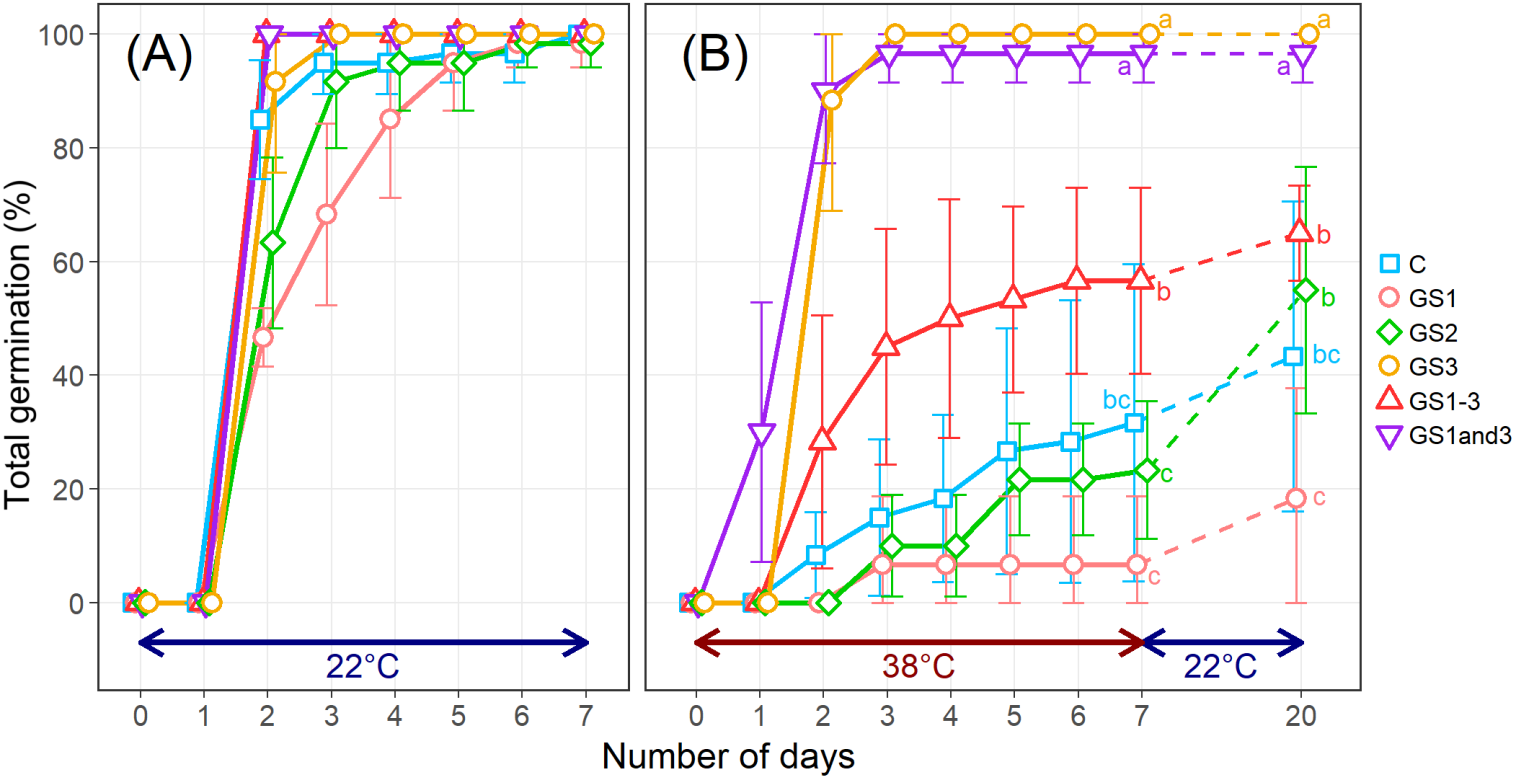
Germination rates of seeds from wheat plants exposed to high temperature at various times during the growing season. **(A)** Germination at 22°C. **(B)** Germination at 38°C. On day 8, the seeds were placed at 22°C for an additional 13 d to investigate whether the ungerminated seeds were dead or in secondary dormancy. Treatment identifiers are as in **Figure 1**. Error bars represent standard deviations. Significances of differences among treatments were tested using analysis of variance and Tukey’s range test, and shared letters indicate lack of significance.

Heat application at different growth stages induced stage-specific morphological changes in seeds, with GS3, GS1–3, and GS1&3 exhibiting significantly reduced seed size, as indicated by a 25.3% decrease in thousand-kernel weight (**Figure 2**, **Supplementary Table S2**). While all seeds germinated at 22°C within seven days, the control seeds had a low germination rate (32%) at 38°C. Seeds from GS1 (7%) and GS2 (23%) showed even lower rates, whereas GS3 (100%) and GS1&3 (97%) had significantly higher rates, and GS1–3 had an intermediate rate of 57% (**Figure 3**). To determine whether seeds that failed to germinate at 38°C had lost viability or entered secondary dormancy, we transferred them to 22°C on 7 DAI. Germination in control seeds increased by 12%, resulting in a 44% heat survival rate. In contrast, the mean germination rate of GS2 increased to 55%, suggesting that GS2 can survive at a higher rate under 38°C compared to the control and GS1.

### 3.2 Heat shock proteins

**Figure 4:**
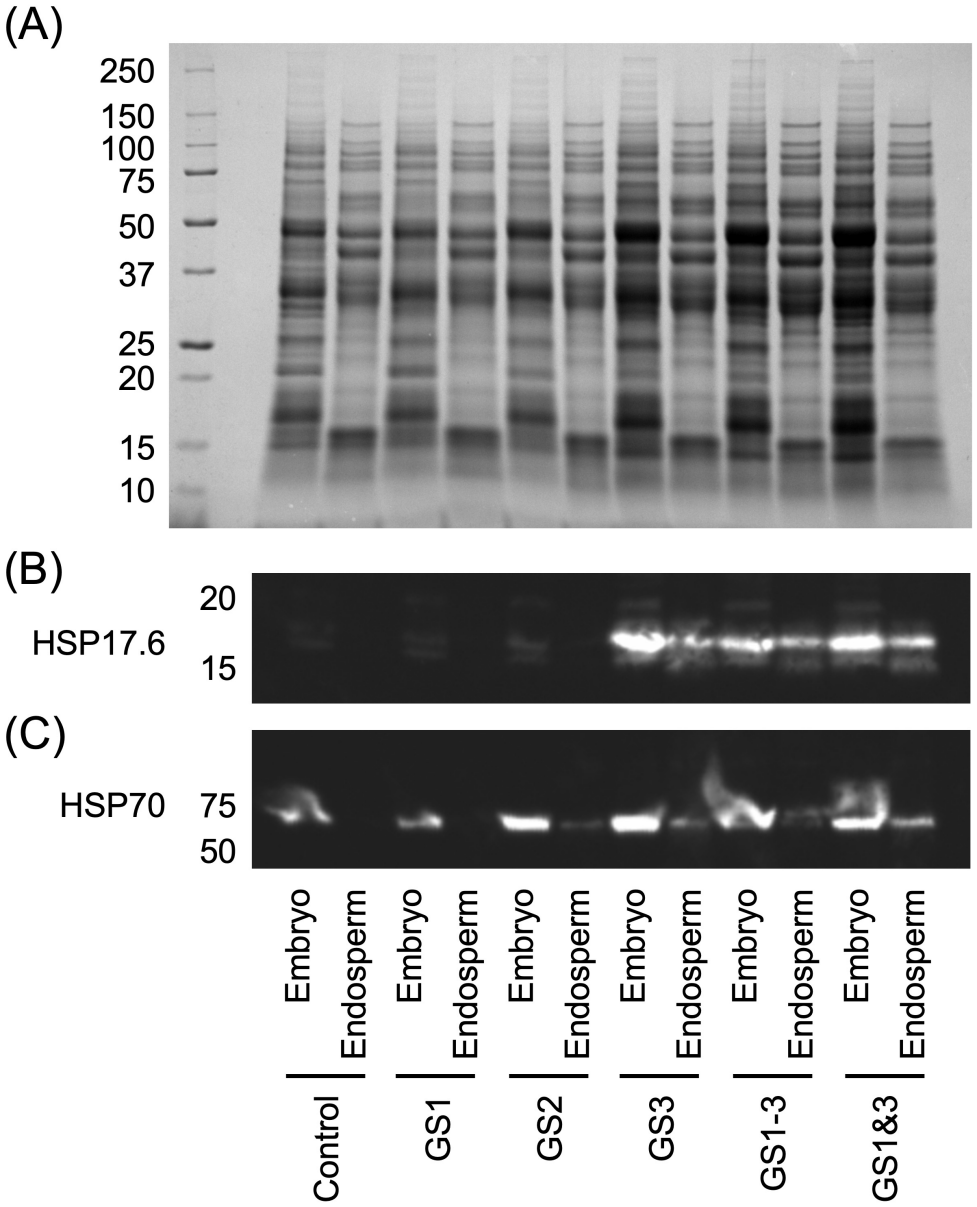
Results of immunoblot analyses of heat shock proteins (HSPs) in embryos and endosperms of wheat seeds from plants grown with heat treatment at various stages of development. **(A)** Sodium dodecyl sulphate-polyacrylamide gel electrophoresis (SDS-PAGE). **(B, C)** Expression levels of HSP17.6 and HSP70, respectively. Treatment identifiers are as in **Figure 1**.

HSP17.6 was detected specifically in seeds from parents treated at high temperature during the seed formation period, i.e., GS3, GS1–3, and GS1&3 (**Figure 4B**). The protein expression levels were higher in the embryo than in the endosperm. GS3 and GS1&3 had higher expression levels than GS1–3. This is consistent with the observed germination rates at high temperature (i.e., heat survival rates; **Figure 3B**), i.e., GS3 ≥ GS1&3 > GS1–3. HSP70 was detected in all embryos (**Figure 4C**), but the levels were lowest in the control and GS1 embryos.

### 3.3 Fatty acid saturation

**Figure 5:**
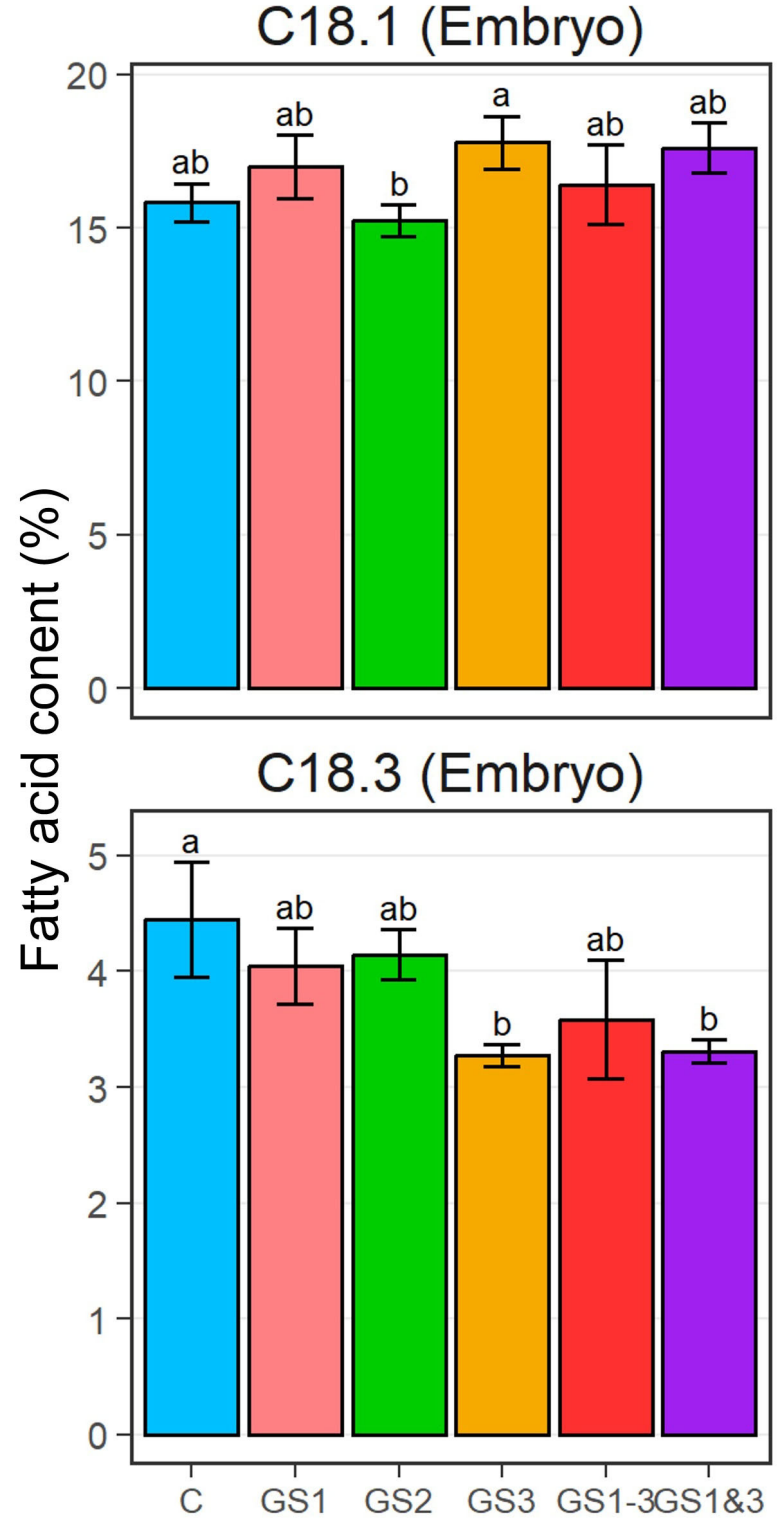
Fatty acid ratio of oleic acid (C18:1; top) and linolenic acid (C18:3; bottom) in embryos of seeds from each heat treatment. Bars and error bars indicate mean and ±1 standard deviation, respectively. Lowercase letters indicate significant differences (*p* < 0.05, by Tukey’s range test). Treatment identifiers are as in **Figure 1**.

For palmitic acid (C16:0), stearic acid (C18:0), and linoleic acid (C18:2) in the seed embryos and endosperms, the differences in fatty acid compositions were not significant among the treatments (**Supplementary Table S3**). However, significant differences were observed in oleic acid (C18:1) and linolenic acid (C18:3) in the embryos (**Figure 5**). The C18:1 content of embryos from the GS2 and GS3 treatments were significantly different. Also, the C18:3 content in embryos from treatments GS3 and GS1&3 were significantly lower than in the control.

### 3.4 Accumulation of metabolites

By the adjustment to counteract the overall trend resulting from the heat treatments, it was found that the seed content of many metabolites appeared unaffected by heat treatments (**Supplementary Figure S1**). Additionally, the log2(FC) transformation of the metabolite contents improved the normality of their distribution (**Supplementary Figure S2A**) and stabilized their variance (**Supplementary Figure S2B**), which justified the use of this transformation.

**Figure 6:**
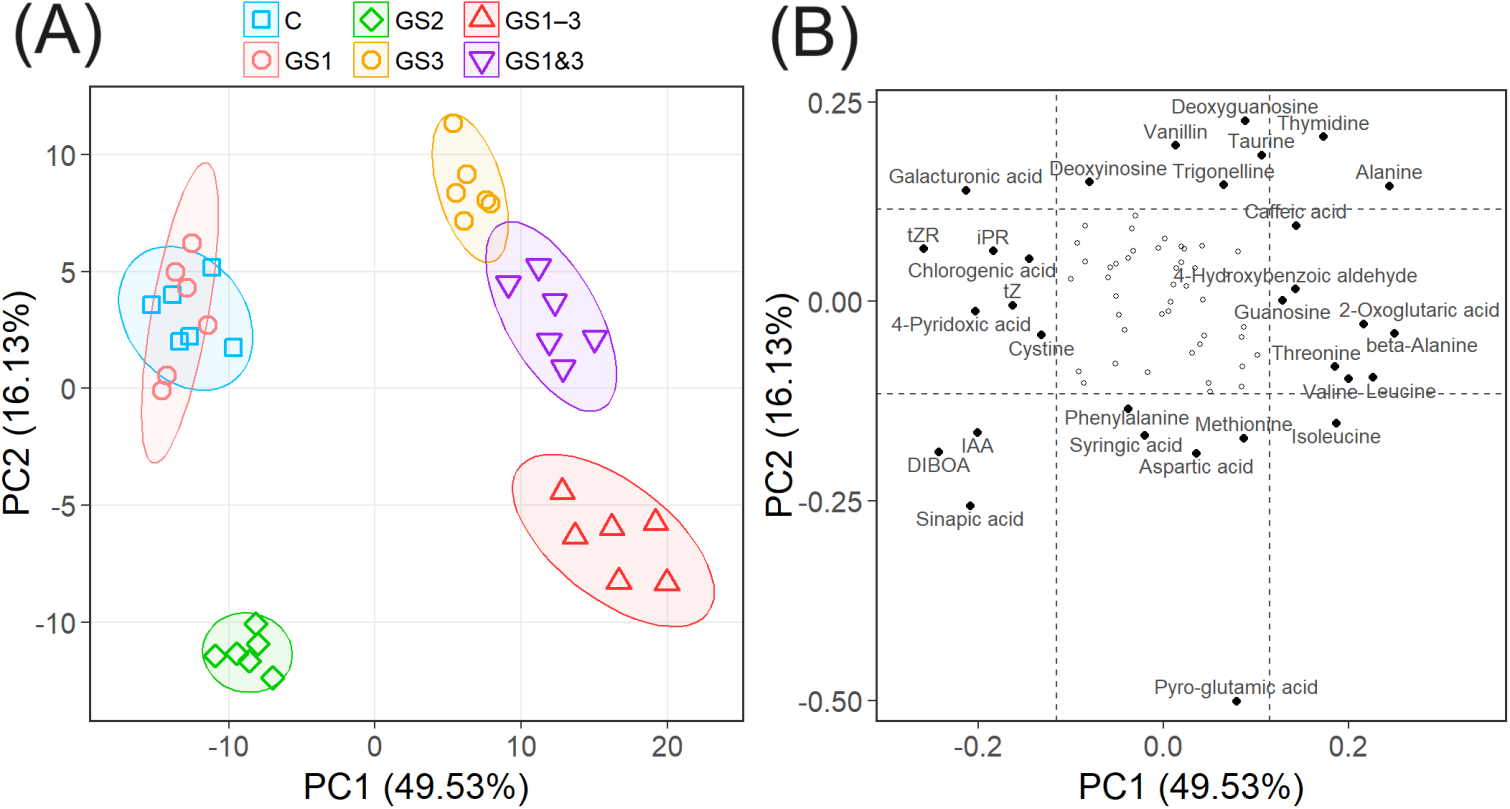
Principal component analysis of metabolite content of seeds from wheat exposed to high temperature at various stages of growth. **(A)** First and second principal components (PC1 and PC2, respectively). Ellipses indicate the range in which the seeds from each heat treatment are included with 90% probability. Treatment identifiers are as in **Figure 1**. **(B)** Loading score of PC1 and PC2. Dotted lines represent ±1 standard deviation from 0, and metabolites outside these ranges are named. Proportions of the variances explained by each principal component are included in the axis titles.

PCA of metabolite contents revealed that the first principal component (PC1) scores correlated with exposure to high temperature during the seed formation period (**Figure 6A**). Samples from plants exposed to such conditions (treatments GS3, GS1–3, and GS1&3) had positive PC1 values, and were distinctly separated from those not so exposed (C, GS1, and GS2). The second principal component (PC2) scores reflected exposure to high temperature from tillering to flowering, and separated samples with this exposure (GS2 and GS1–3) from the remainder (**Figure 6A**). The two remaining principal components (PC3 and PC4) were not clearly associated with the heat treatments (**Supplementary Figure S3**).

**Figure 7:**
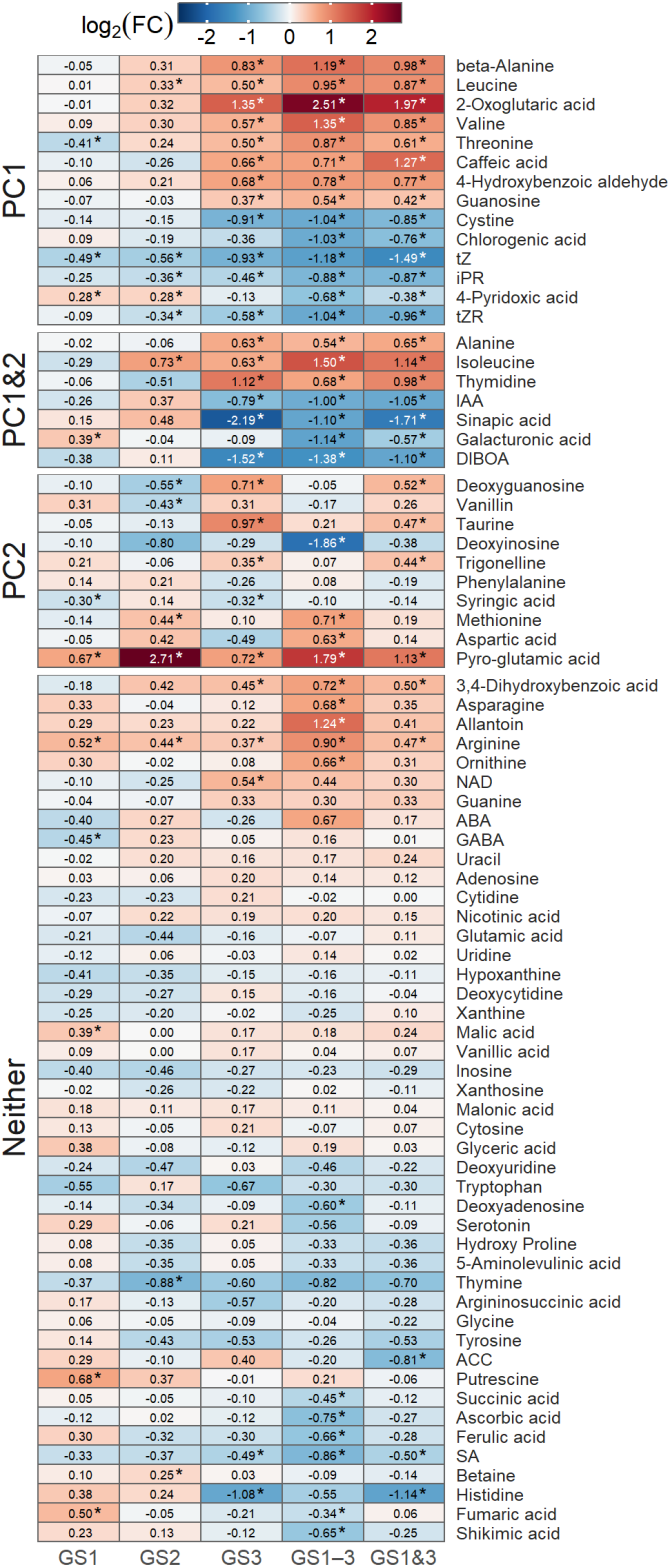
Heatmap of logarithms of fold changes in metabolite content [Log_2_(FC)] compared to the control, in seeds from plants exposed to high temperature at various growth stages. Asterisks indicate significant differences (*p* < 0.01) from the control treatment, using Dunnett’s method. Groupings indicate whether the metabolite had large loading scores of PC1 or PC2 (see **Figure 6**). Treatment identifiers are as in **Figure 1**.

The phytohormones such as auxin (IAA) and cytokinins (riboside precursor of isopentenyladenine (iPR), transzeatin (tZ), and its riboside precursor (tZR)), amino acids, and organic acids showed large loading scores of PC1. The seed content of most of these metabolites differed significantly (*p* < 0.01) from the control (**Figure 7**), which were either increased or decreased substantially in seeds from plants exposed to heat treatment during seed formation (GS3, GS1–3, and GS1&3). Pyro-glutamic acid had the largest effect on PC2, followed by several other metabolites including organic acids, nucleic acids, and amino acids (**Figure 6B**). Among these, the seed content of pyro-glutamic acid increased markedly in GS2 and GS1–3. Many of the metabolites (45 out of 76) were not related to PC1 or PC2, and differed little among the heat treatments.

The coefficients of determination of PLS regression were 0.424 on the heat germination rate and 0.452 on the heat survival rate under heat. Metabolites with top 10 absolute values of these regression coefficients corresponded to those which had high impact on PC1 and PC2 (**Table 1**).

**Table 1:** Coefficients of partial least squares (PLS) regression, one of the methods of linear regression robust to multicollinearity, on the heat germination rate and heat survival rate of wheat seeds exposed to heat during germination (10 highest absolute values only).

Germination rate under heat			Survival rate under heat		
Metabolite	Related PC	Coef.	Metabolite	Related PC	Coef.
Taurine	2	0.0947	Galacturonic acid	1&2	1.23
Sinapic acid	1&2	−0.0847	beta-Alanine	1	1.03
Alanine	1&2	0.0772	Valine	1	1.02
Thymidine	1&2	0.0756	Sinapic acid	1&2	−0.806
Caffeic acid	1	0.0723	Thymidine	1&2	0.788
Deoxyguanosine	2	0.0696	Threonine	1	0.716
4-hydroxybenzoic aldehyde	1	0.0661	Deoxyguanosine	2	0.701
Guanosine	1	0.0631	Taurine	2	0.657
beta-Alanine	1	0.0616	4-Pyridoxic acid	1	0.607
DIBOA	1&2	−0.0610	Aspartic acid	2	−0.573

The related principal components (PC) are based on their Log_2_(FC) loadings as shown in **Figures 6**.

## 4 Discussion

### 4.1 Seed development and germinability

The study reported here found that heat exposure during specific growth stages significantly impacted germination rates including heat survival rates at high temperature, exhibiting diverse patterns. The greatest impact was associated with heat during the seed formation, resulting in a significant increase in rates of germination at elevated temperature compared with control. For some treatments, germination rate at 38°C was more than twice that of the control (**Figure 3**). In lettuce, high temperatures during seed formation elevate both the germinable temperature and the germination rate of the seeds (Kozarewa et al., 2006), but a similar phenomenon has not been intensively studied in cereal crops.

Among treatments including heat during the seed formation period, the GS3 and GS1&3 heat treatments resulted in near 100% germination rate at the high temperature, whereas the seeds from treatment GS1–3 had a much lower high-temperature germination rate. The lower germination rate in GS1–3 is likely attributable to the prolonged exposure to heat, which inhibited the growth of the parental plant and resulted in a deficiency in the seeds of the metabolites that are needed for germination at high temperatures. Kranner et al. (2010) proposed a model for stress responses in plant seeds, which consists of three phases: ‘alarm’, ‘resistance’, and ‘exhaustion’, in increasing order of stress severity. According to this model, the seeds from GS3 and GS1&3 in this experiment can be interpreted as having acquired ‘resistance’, while the seeds from GS1-3 lost heat germinability due to ‘exhaustion’.

The heat germinability of GS2 seeds was lower than that of the control seeds. Since there are two causes for lack of germination at high temperature, death and secondary dormancy (Baskin and Baskin, 2014), the improved germination of GS2 seeds when transferred to 22°C after initial exposure to 38°C during germination indicated that they were likely in secondary dormancy. This suggests that the GS2 treatment may provide seeds with some high-temperature tolerance, though weaker than the GS3 and GS1&3 treatments, and that seeds produced in GS2 growing conditions may not germinate at high temperature but instead shift to secondary dormancy.

The differences in primary dormancy depth among individuals and treatments are considered to have had minimal impact on the results of this experiment. There are reports indicating that the depth of dormancy can vary depending on the temperature conditions during seed formation (Reddy et al., 1985), as well as reports suggesting significant individual variation in dormancy depth (Nyachiro et al., 2002). However, in this study, even the control seeds, which were expected to have the deepest primary dormancy among all treatments, showed a 100% germination rate within 7 days under the 22°C germination conditions (**Figure 3A**). Additionally, the variation in germination rates among individuals and treatments was very small. This suggests that the methodology used in this experiment successfully broke primary dormancy and minimized its influence.

### 4.2 Heat shock proteins

Both HSP17.6 and HSP70 have been reported to contribute to the improvement of heat tolerance, by maintaining cell function and in protein repair (Blumenthal et al., 1998; Hahn et al., 2011; Rikhvanov et al., 2007), but their roles in heat tolerance acquisition of seeds are unknown.

HSP17.6 was detected in the seeds with high germinability in the high-temperature environment (GS3, GS1–3, and GS1&3), which showed high germination rate under heat. Since the late 1980’s, a large body of evidence has been accumulated to support the concept that small HSPs, in particular HSP17.6, accumulate at the late stage of seed development (and their accumulation increases with temperature) (Bettey and Finch-Savage, 1998; Sun et al., 2001; Wehmeyer et al., 1996). They are involved in protein unfolding and refolding during seed desiccation and imbibition and are directly connected to seed vigor. Bettey and Finch-Savage (1998) reported that accumulation of HSP17.6 in seeds during seed filling enhanced stress tolerance in subsequent germination in cabbage. A previous study in Arabidopsis found that HSP17.6 was expressed in cultured cells exposed to moderately high temperatures (37°C), resulting in the acquisition of tolerance to excessively high temperatures (50°C; Rikhvanov et al., 2007). However, this report only investigated the behavior during a few hours, and there are no reports of the persistence of this heat tolerance from one generation to the next through seed. In the study reported here, high temperature treatment of the parental generation induced expression of HSP17.6 in its seeds, i.e., the treatment apparently conferred high temperature germination ability.

HSP70 was detected at higher levels in embryos of GS2, GS3, GS1–3 and GS1&3 than in those of control and GS1. A common feature of these seeds was that they had high heat survival rates in the high-temperature conditions. This suggests that heat exposure after tillering may have induced expression of HSP70 in embryos, thereby increasing the heat survival ability of seeds produced in high-temperature environments. It is interesting to note that HSP70 proteins were up-regulated in embryos from plants that experienced heat during GS2, regardless of the normal temperature during grain development. Apparently, the effect of high temperature during initial anther and pistil development may be retained during post-fertilization development.

HSP70 was detected mainly in the embryo, whereas HSP17.6 was detected in both the embryo and the endosperm. This difference is consistent with the above discussion of specific roles of HSPs. While HSP17.6 enhanced the heat germination rate of the entire seed, HSP70 may have been responsible for enhancing the heat survival rate by being expressed in the embryo.

Together, our results suggest that the high temperature during seed formation induced specific expression of HSPs, which may have contributed to protein repair and maintenance of cell function under heat exposure. These mechanisms are thought to also result in germination tolerance to high temperature. Future experiments with transformants may further elucidate the effects and mechanisms of HSPs.

### 4.3 Fatty acids

The two fatty acids which showed significant difference in their contents, C18:1 and C18:3, trended in opposite directions, in comparison with the controls, i.e., in both GS3 and GS1&3, C18:1 increased while C18:3 decreased. This suggests that heat exposure during seed formation may have induced the replacement of C18:3 with C18:1, which represents an increase of saturation of the C18 fatty acid, to maintain biomembrane structural stability at high temperatures. These changes were similar to those reported in wheat leaves at high temperatures during seed formation (Narayanan et al., 2016), which suggests that the entire plant may acquire heat tolerance through fatty acid remodeling. However, although plants in the GS1–3 treatment were also exposed to heat during seed formation, fatty acid composition of GS1–3 seeds did not differ considerably from that of the control seeds. Prolonged exposure to heat influences plant growth in many different ways (Matsunaga et al., 2021a, 2021b). Acquisition of sufficient heat tolerance by GS1–3 seeds may have been inhibited by the prolonged exposure to heat, which may be one of the reasons for the inferior germinability at high temperatures of GS1–3 compared to GS3 and GS1&3. These results suggest that the changes in membrane structure attributable to high temperature around the period of seed formation reduced the fluidity of biological membranes at high temperature.

### 4.4 Plant hormones

Among phytohormones, auxin (IAA) had a particularly strong effect on PC1 but also on PC2 (**Figure 6B**). The seed content of auxin was significantly lower than the control in treatments with heat during the seed formation period (GS3, GS1–3, and GS1&3, **Figure 7**). In sunflower embryos, auxin inhibits the expression of small HSP transcription factors (Carranco et al., 2010). The concurrence of reduced auxin content and detection of HSP17.6 in seeds suggests that auxin may be involved in the acquisition of germinability under heat, via expression of HSP17.6. Additionally, it has been reported in Arabidopsis studies that IAA affects seed germination through interactions with other plant hormones, such as gibberellin, ethylene, and ABA (Brady et al., 2003; Ogawa et al., 2003), suggesting that various possibilities need to be considered.

Cytokinin is responsible for the regulation of plant growth and senescence (Hönig et al., 2018). Cytokinins isopentenyladenine (iP) and transzeatin (tZ) differ in the structure of the side chain added to the adenine moiety. In this study, tZ, its riboside precursor (tZ riboside, tZR), and the riboside precursor of iP (iP riboside, iPR) were detected. Seed contents of tZ almost halved in treatments GS3, GS1–3, and GS1&3, suggesting that the germination rate at high temperature may be improved when the cytokinin content is reduced. It was reported that the functional loss of cytokinin receptors accelerates germination (Riefler et al., 2006). Also, the decrease of the seed contents of iPR and tZR accompanied the accumulation of HSP70 in the embryos in our study. Berka et al. (2022) focused on the changes in metabolites after imbibition of Arabidopsis seeds, and found that cytokinin up-regulates HSP70. More detailed examination of changes in cytokinins in seeds is needed to elucidate its effect on germinability under heat.

ABA is one of the key plant hormones that regulate seed germination. The presence of ABA maintains seed dormancy, while its degradation leads to dormancy release (Rodríguez-Gacio et al., 2009). In this experiment, as described in Section 4.1, the differences in germination speed among treatments under the 22°C germination condition were minimal (**Figure 3A**), suggesting that the depth of primary dormancy was nearly uniform. This is further supported by the lack of significant differences in seed ABA content among treatments.

### 4.5 Other metabolites

Few studies have analyzed the effects of high-temperature stress during the seed development stage in wheat from a metabolic perspective. However, the findings of a previous study on amino acids (Dias et al., 2008) were largely consistent with the results of this study, especially for threonine and histidine.

Focusing on the dominant trends among metabolites, the seed content of many metabolites was influenced by heat stress during the seed development stage, as reflected in PC1 (**Figure 7**). Among these, sinapic acid, thymidine, caffeic acid, and beta-alanine exhibited particularly high PLS regression coefficients (**Table 1**). Exogenous sinapic acid regulates germination in Arabidopsis by antagonizing ABA; concentrations above 2 mM inhibit germination, while lower concentrations promote it (Bi et al., 2017). Thymidine is a deoxynucleotide synthesized through both the *de novo* and salvage pathways (Xu et al., 2015), and its salvage pathways play a crucial role in the transition of Arabidopsis to autotrophy after germination (Pedroza-García et al., 2019). Caffeic acid plays a role in stress mitigation in plants, contributing to drought and salinity tolerance through its antioxidant activity and enhancing tolerance to pathogen and heavy metal stress by inducing lignin accumulation (Riaz et al., 2018). Proteins involved in beta-alanine metabolism are specifically altered in wheat seeds exposed to drought and salt stress during germination, potentially contributing to stress responses (Yan et al., 2021). Additionally, beta-alanine has been identified as a metabolic marker of the heat priming effect in wheat (Matsunaga et al., 2021a). However, it remains unclear which of these metabolites contributed to the improvement in heat germinability or whether they had any effect at all.

Among the metabolites contributing to PC2, some (taurine, deoxyguanosine, and trigonelline) exhibited a characteristic trend in which their seed content in the GS1–3 treatment, despite experiencing high temperatures during the seed development stage, approached that of the control treatment (**Figure 7**). This trend aligns with the ‘resistance’, and ‘exhaustion’ phases of the stress model proposed by Kranner et al. (2010), as mentioned in Section 4.1. Given that GS1–3 exhibited reduced germination rates due to prolonged high-temperature stress during the growth period, these metabolites may have been particularly involved in both the acquisition and failure of heat germinability.

Among them, taurine and deoxyguanosine had notably high PLS regression coefficients (**Table 1**). Taurine has antimutagenic activity *in vitro* comparable to other major antioxidants (Sung et al., 1999). During germination of bread wheat, it protects cell membranes by promoting early growth and reducing membrane lipid peroxidation products (Hao et al., 2004). Thus, taurine may have improved germinability in heated conditions, through antimutagenic activity and/or protection of membrane lipids. Kuo et al. (2004) reported that the taurine content in lentils increases with days after imbibition during germination in light, demonstrating a relationship between taurine and germination.

Deoxyguanosine is associated with DNA damage from ROS. ROS induce the transformation of guanine in DNA to 7,8-dihydro-8-oxoguanine, which binds with deoxyguanosine and forms 8-hydroxy-2′-deoxyguanosine (Chen et al., 2012), which is a biomarker for oxidative stress. Low deoxyguanosine content may indicate the severity of DNA damage. In this study, the relative seed contents of deoxyguanosine among treatments were GS3 = GS1&3 > C = GS1 = GS1–3 > GS2. The relatively high seed content of deoxyguanosine in GS3 and GS1&3 suggests less DNA damage in the seeds, which may have contributed to higher heat germination and heat survival rates at the high temperature. The relatively low deoxyguanosine content in GS2, which may indicate relatively high DNA damage, could be attributable to high temperatures during the stress-sensitive juvenile spike formation stage. This may account for the inferior heat germination and survival rates compared to the control, despite the expression of HSP70 in the embryos of GS2 seeds.

Lastly, pyro-glutamic acid exhibited a distinct pattern of seed content compared to other metabolites. The seed content of pyro-glutamic acid differed greatly from the control in heat treatments from tillering to flowering (GS2 and GS1–3; **Figure 7**). The contribution of pyro-glutamic acid to germination and yield enhancement have been patented (Unkefer et al., 2007). The specific mechanism(s) of pyro-glutamic acid in germinability remain unreported, and perhaps unidentified.

Many metabolites were likely related to the acquisition of seed germinability during high temperatures within the growth period of parental plants. Although the roles of specific metabolites have been discussed, germinability is a complex phenotype affected by the interactions of multiple factors. Their combined effects will need to be assessed further to elucidate the mechanisms by which germinability at high temperatures is acquired. Comparisons of the content of these metabolites may become useful in selecting lines that show high-temperature tolerance during germination. Similar analyses using seeds grown in different environments will also be needed to verify whether these markers are versatile.

## 5 Conclusions

Exposure to high temperature at specific growth stages had diverse effects on germination and seed survival ability under heated conditions. The findings here may represent a significant step forward in understanding the mechanisms underlying the acquisition of heat tolerance in wheat seeds. The phenomena observed in this study may not be directly applicable in breeding and cultivation since they occurred after exposure to elevated temperature during growth. Nevertheless, the possibility that seeds from different environments may exhibit different heat tolerances, despite sharing the same genotype, has the potential to better inform cultivation practices. For example, the importation of seeds from a cool region to a hot region may result in a lower germination rate than anticipated, due to the seeds’ inherent low heat tolerance. Further elucidation of this mechanism of heat tolerance acquisition could enable artificial enhancement of seed heat tolerance by using key metabolites and/or phytohormones. This may become more important as climate change continues, because the period during which seeds can germinate and the period of high temperatures will overlap with greater frequency in the future. The findings of this study suggest the potential for using seed heat tolerance in the enhancement of wheat production.

## Data Availability

The raw data supporting the conclusions of this article will be made available by the authors, without undue reservation.
