# Supplementary material for: Wheat seeds exposed to heat during formation can germinate at high temperatures
Source: Front Plant Sci. 2025 Mar 28;16:1539926. doi: 10.3389/fpls.2025.1539926 (PMC11985767; doi:10.3389/fpls.2025.1539926)
Supplement: Supplementary file 1 [file DataSheet1.docx]

Supplementary Material

**Appendix**. Simulation of germination test.

A simulation study was conducted to validate whether the number of seeds in our experiment ensures the reproducibility of the germination test results. The germination rate under the 38°C condition, 7 days after imbibition, was chosen as the simulation target. The mean germination rates for each treatment (31.7%, 6.67%, 23.3%, 100%, 56.7%, and 96.7% for treatments C, GS1, GS2, GS3, GS1–3, and GS1&3, respectively) were set as the true values. The number of germinated seeds was randomly generated for each parent plant, following a binomial distribution. The number of parent plants was set to 6, as in the experiment, and the number of seeds per parent plant varied between 5 and 30. The simulation was repeated 1,000 times for each of the number of seeds. Analysis of variance and Tukey's range test were applied to the simulated results, validating whether the experimental results were reproducible—that is, whether the same differences among treatments (six differences: GS3 > GS1–3, GS1&3 > GS1–3, GS3 > C, GS1&3 > C, GS1–3 > GS2, and GS1–3 > GS1) could be detected significantly (*p* < 0.05). As a result, the percentage of correct detections reached 93.1% when the number of seeds was 10 (Figure S4). This indicates that, despite the relatively limited number of seeds, the results from our experimental design are highly reproducible.

**Supplementary Table 1.** Reaction monitoring conditions for detected metabolites.

See “TableS1_Matsunaga.xlsx”.

**Supplementary Table 2**. Yield-related traits of plants exposed to heat treatments during growth. The means, followed by standard deviations in parentheses, of thousand kernel weight (TKW, g), the number of grains, and yield (g) are presented. Differences among treatments were analyzed using analysis of variance and Tukey’s range test. Significant differences (*p* < 0.05) are indicated with superscript letters.

| Treatment | TKW (g) | Num. of grains | Yield (g) |
| --- | --- | --- | --- |
| C | 33.3 (1.22)^b^ | 784 (101)^a^ | 26.1 (3.58)^a^ |
| GS1 | 38.3 (2.07)^a^ | 756 (339)^a^ | 28.5 (11.88)^a^ |
| GS2 | 35.2 (1.22)^ab^ | 573 (76)^ab^ | 20.1 (2.67)^ab^ |
| GS3 | 27.0 (0.80)^c^ | 703 (127)^ab^ | 19.0 (3.75)^ab^ |
| GS1–3 | 26.8 (3.86)^c^ | 418 (39)^b^ | 11.2 (1.45)^b^ |
| GS1&3 | 20.8 (0.95)^d^ | 697 (197)^ab^ | 14.6 (4.27)^b^ |

**Supplementary Table 3.** Content of five different fatty acids in embryos and endosperm of seeds from different heat treatments during growth of the parent plants, as a percentage of the total content of all five. Values are means ± standard deviation. Treatment identifiers are as in Figure 1.

| Treatment | Tissue | C16:0 | C18:0 | C18:1 | C18:2 | C18:3 |
| --- | --- | --- | --- | --- | --- | --- |
| Control | Embryo | 20.15 ±0.78 | 2.78 ±1.26 | 15.82 ±0.63 | 56.80 ±1.73 | 4.45 ±0.50 |
| GS1 | Embryo | 19.48 ±1.49 | 2.98 ±0.89 | 16.99 ±1.03 | 56.51 ±2.54 | 4.05 ±0.33 |
| GS2 | Embryo | 19.60 ±0.89 | 2.53 ±0.85 | 15.24 ±0.51 | 58.48 ±2.13 | 4.15 ±0.22 |
| GS3 | Embryo | 21.44 ±1.49 | 3.41 ±0.75 | 17.78 ±0.87 | 54.09 ±0.31 | 3.28 ±0.09 |
| GS1–3 | Embryo | 22.57 ±4.19 | 2.90 ±0.61 | 16.41 ±1.29 | 54.52 ±3.61 | 3.59 ±0.51 |
| GS1&3 | Embryo | 20.72 ±0.34 | 3.11 ±0.71 | 17.61 ±0.80 | 55.24 ±1.52 | 3.31 ±0.10 |
| Control | Endosperm | 23.34 ±0.72 | 3.74 ±0.85 | 13.32 ±1.59 | 56.42 ±2.50 | 3.18 ±0.09 |
| GS1 | Endosperm | 22.83 ±2.46 | 3.85 ±1.03 | 13.55 ±1.37 | 56.51 ±3.27 | 3.26 ±0.32 |
| GS2 | Endosperm | 24.62 ±0.63 | 4.18 ±0.96 | 11.19 ±1.52 | 56.67 ±2.00 | 3.35 ±0.40 |
| GS3 | Endosperm | 25.34 ±1.49 | 5.05 ±0.98 | 12.27 ±0.22 | 54.58 ±0.38 | 2.77 ±0.11 |
| GS1–3 | Endosperm | 25.33 ±2.51 | 5.35 ±1.27 | 11.48 ±1.78 | 54.81 ±2.72 | 3.04 ±0.54 |
| GS1&3 | Endosperm | 24.45 ±0.41 | 5.17 ±1.17 | 12.25 ±0.71 | 55.26 ±2.15 | 2.87 ±0.12 |


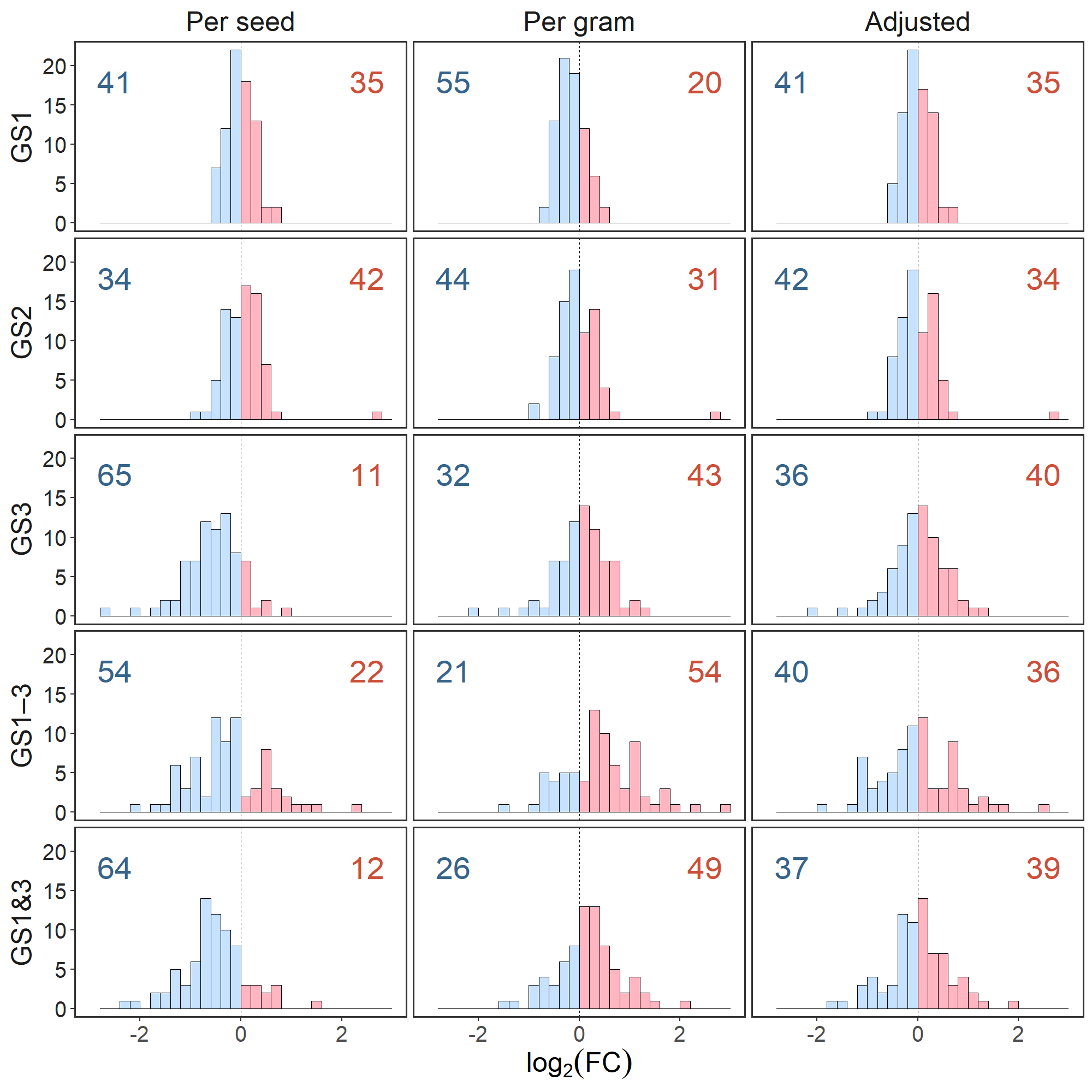


**Supplementary Figure 1.** The distributions of base 2 logarithms of mean fold changes (FC, value for the metabolite in the seed from a heat treatment divided the corresponding value for the control) for three adjustment methods for metabolite content of seed from wheat grown under various heat treatments. (A) Calculated per seed. (B) Calculated per gram. (C) Adjusted for overall trend. Blue and orange numbers indicate the number of metabolites for which the mean log_2_(FC) was negative and positive, respectively. Treatment identifiers are as shown in Figure 1.


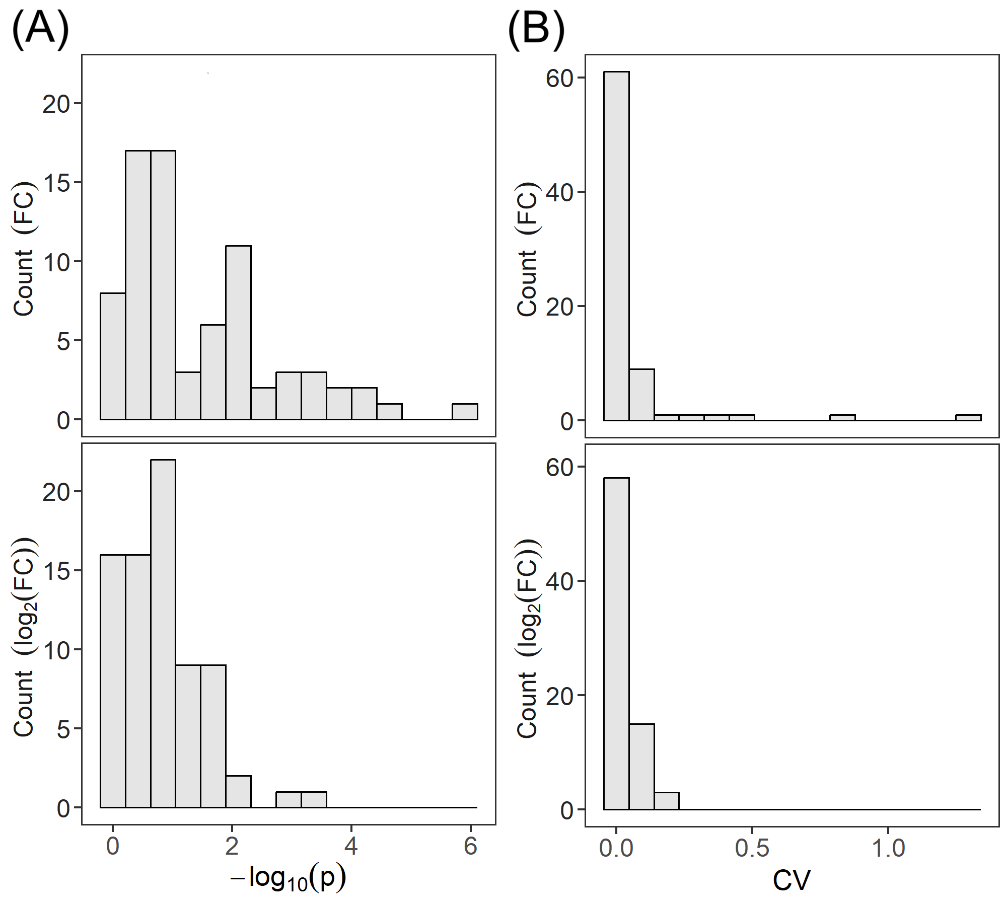


**Supplementary Figure 2.** Summary statistics of deviations of fold changes (FC) and log_2_(FC) of adjusted seed metabolite contents from the treatment means. (A) Distributions of −log_10_(*p*) of Shapiro–Wilk test applied to the deviations for each metabolite. Larger −log_10_(*p*) values indicate that the distribution of the deviances is not a normal distribution. (B) Distributions of coefficients of variation (CV) of variances of the deviations. The variances of the deviations were calculated for each treatment and metabolite, and then CVs of the variances were calculated for each metabolite. Larger CV values indicate that the variance of the deviation was not consistent among treatments. The sum of the counts in each graph equals the number of measured metabolites.


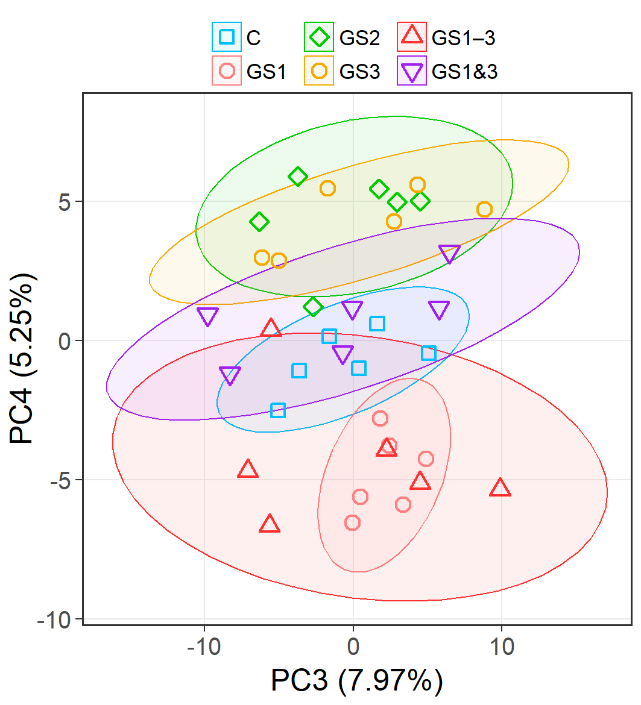


**Supplementary Figure 3.** Third and fourth principal components (PC3 and PC4, respectively) of metabolite composition of seeds from wheat exposed to high temperature at various stages of growth. Treatment identifiers are as shown in Figure 1. Ellipses are as described in Figure S2A. Proportions of the variances explained by each principal component are included in the axis labels.


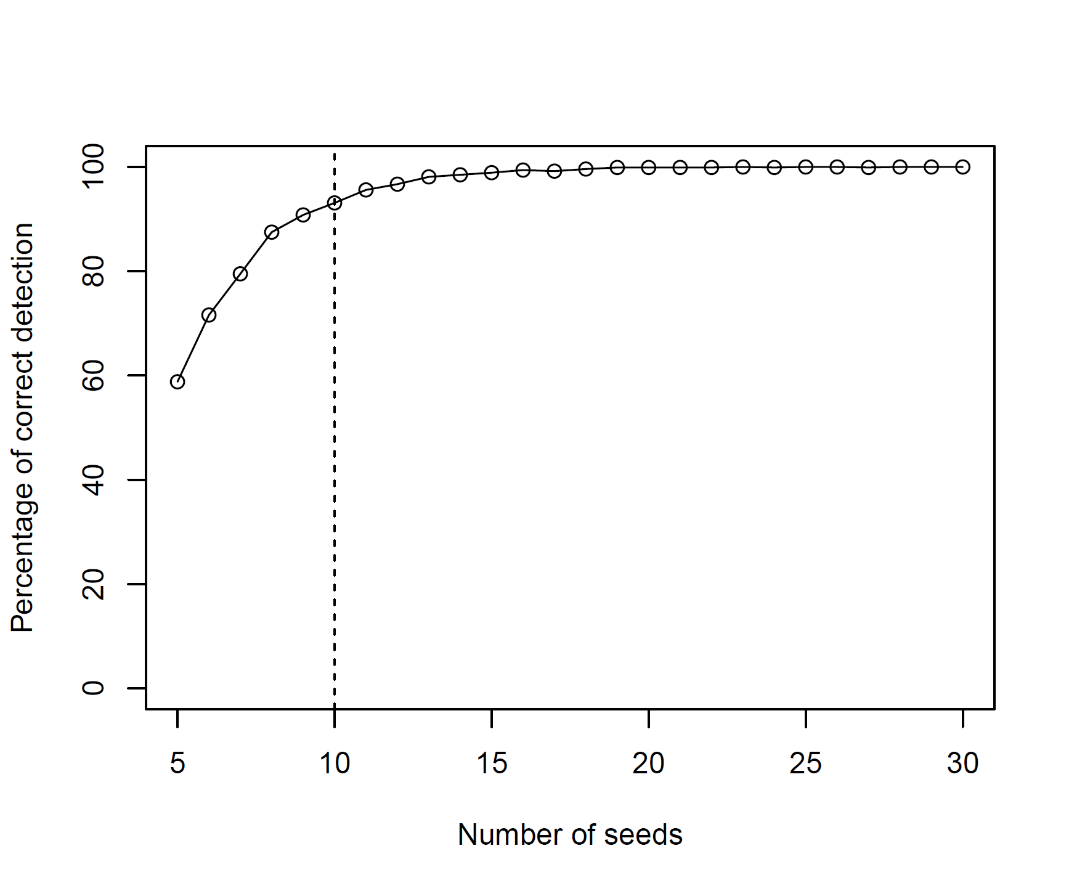


**Supplementary Figure 4**. Result of a simulation of the germination test. The relationship between the number of seeds per parent plant and the percentage of correct detections is illustrated. The vertical dotted line represents the number of seeds used in the experiment.
